# Long-Term Effects of Traumatic Brain Injury on Anxiety-Like Behaviors in Mice: Behavioral and Neural Correlates

**DOI:** 10.3389/fnbeh.2019.00006

**Published:** 2019-01-23

**Authors:** Juliana Popovitz, Shreesh P. Mysore, Hita Adwanikar

**Affiliations:** ^1^Department of Psychological and Brain Sciences, Johns Hopkins University, Baltimore, MD, United States; ^2^Department of Neuroscience, Johns Hopkins University, Baltimore, MD, United States

**Keywords:** traumatic brain injury, anxiety behaviors, basolateral amygdala, controlled cortical impact, GABA

## Abstract

Traumatic brain injury (TBI) has been frequently linked to affective disorders such as anxiety and depression. However, much remains to be understood about the underlying molecular and signaling mechanisms that mediate affective dysfunctions following injury. A lack of consensus in animal studies regarding what the affective sequelae of TBI are has been a major hurdle that has slowed progress, with studies reporting the full range of effects: increase, decrease, and no change in anxiety following injury. Here, we addressed this issue directly by investigating long-term anxiety outcomes in mice following a moderate to severe controlled cortical impact (CCI) injury using a battery of standard behavioral tests—the open field (OF), elevated zero maze (EZM), and elevated plus maze (EPM). Mice were tested on weeks 1, 3, 5 and 7 post-injury. Our results show that the effect of injury is time- and task-dependent. Early on—up to 3 weeks post-injury, there is an increase in anxiety-like behaviors in the elevated plus and zero mazes. However, after 5 weeks post-injury, anxiety-like behavior decreases, as measured in the OF and EZM. Immunostaining in the basolateral amygdala (BLA) for GAD, a marker for GABA, at the end of the behavioral testing showed the late decrease in anxiety behavior was correlated with upregulation of inhibition. The approach adopted in this study reveals a complex trajectory of affective outcomes following injury, and highlights the importance of comparing outcomes in different assays and time-points, to ensure that the affective consequences of injury are adequately assessed.

## Introduction

Traumatic brain injury (TBI), characterized as any damage to the brain caused by external acceleration or deceleration forces (Ingebrigtsen and Romner, 2003; Menon et al., 2010), is a complex health problem affecting millions of people worldwide (Hyder et al., 2007). TBI produces considerable and wide-ranging losses in cognitive, motor and affective functions (Draper and Ponsford, 2008; Ponsford et al., 2008). This is true even of injuries considered mild or moderate, which constitute 80% of all cases and can lead to debilitating long-term effects (Buck, 2011; Coronado et al., 2015). The high prevalence and substantial impact of TBI emphasize the importance of understanding the neural mechanisms underlying the outcomes of injury.

Animal models of TBI, and specifically rodent models, have been used to replicate the human symptomatology, examine neural mechanisms and test therapeutic interventions (Xiong et al., 2013). The cognitive and motor outcomes of TBI have been well established and replicated among pre-clinical studies (Fujimoto et al., 2004; Morales et al., 2005; Shear et al., 2010). However, affective outcomes, and specifically, maladaptive anxiety outcomes, which can affect up to 70% of all TBI patients (Rao and Lyketsos, 2002; Moore et al., 2006; Osborn et al., 2016; Scholten et al., 2016), have been difficult to reproduce in animal models. Studies in rodents, which typically quantify anxiety as the proportion of time animals spend in a more exposed, anxiogenic portion of a behavioral apparatus, as opposed to an enclosed, less anxiogenic zone, have yielded inconsistent and, at times, contradictory results (Wagner et al., 2007; Wakade et al., 2010; Schultz et al., 2011; Ajao et al., 2012; Cope et al., 2012; Washington et al., 2012; Budinich et al., 2013; Almeida-Suhett et al., 2014; Amorós-Aguilar et al., 2015; Sierra-Mercado et al., 2015).

One group of pre-clinical studies found an increase in anxiety-like behaviors following TBI. Injured rats and mice spent less time than sham controls in the anxiogenic zones in the open field (OF) test, elevated plus maze (EPM), elevated zero maze (EZM) and in the dark-light chamber tests, and exhibited increased immobility in the tail suspension test for up to 8 weeks post-TBI (Wagner et al., 2007; Schultz et al., 2011; Ajao et al., 2012; Cope et al., 2012; Almeida-Suhett et al., 2014). Paradoxically, another group of studies found a decrease in anxiety-like behaviors, with injured animals spending more time than sham controls in the anxiogenic zones in the EPM and OF tests for up to 3 weeks post-TBI (Wakade et al., 2010; Washington et al., 2012; Budinich et al., 2013). Finally, a third group of studies found no difference between injured and sham animals in the EPM and OF tests for up to 6 weeks post-TBI (Schultz et al., 2011; Amorós-Aguilar et al., 2015; Sierra-Mercado et al., 2015). It is likely that the diversity in range of time-points tested—ranging from 3 to 8 weeks; and differences in the choice of behavioral tests administered to assess anxiety account for these variations. Together, they contribute to the idiosyncratic results reported in the literature. An additional factor that can add to variation is the degree of injury severity, which differs between studies. However, even for a similar injury level, variable results have been described. Wagner et al. (2007), Ajao et al. (2012) and Almeida-Suhett et al. (2014) produced mild controlled cortical impact (CCI) in rats and found increased anxiety at 7 days (Almeida-Suhett et al., 2014), at 14 days (Wagner et al., 2007) and up to 60 days (Ajao et al., 2012). However, Amorós-Aguilar et al. (2015) produced a mild CCI in rats and found no effects on behavior. A moderate to severe CCI can lead to no anxiety early after injury (Sierra-Mercado et al., 2015), increased anxiety at 45 days (Thau-Zuchman et al., 2019), or variable effects depending on the test used (Tucker et al., 2017). Washington et al. (2012) explicitly tested this by using varying levels of injury severity. While severity impacted lesion volume differentially, behavioral effects at 3 weeks after injury were similar. Mild, moderate or severe injury leads to reduced anxiety in the EPM at 21 days post injury, and showed no effects in the OF test. However, moderate or severe injury showed greater lesion volumes and decreased ipsilateral hippocampal volumes, as compared to mild injury.

The primary goal of this study was to explicitly address the issues of variability in time-points and behavioral assays in mice. We hypothesize that there is a time and test dependance of anxiety-like behaviors measured following CCI. To achieve this goal, we used a well-established model of brain injury—the CCI model (Adwanikar et al., 2011; Xiong et al., 2013), employed a battery of commonly used tests to measure anxiety-like behaviors in mice, and measured anxiety over a long time-course. Anxiety behaviors were assessed every 2 weeks up to 7 weeks following moderate to severe injury, and all mice were subjected to a battery of OF, EPM, and EZM tests of anxiety at each time point. This experimental design permitted the direct comparison of different anxiety metrics within the same animals and over time, allowing for a systematic dissection of the behavioral affective sequelae of anxiety following injury. Repeated measures of anxiety tests, on one hand have been interpreted as leading to habituation in response. However, this is also associated with changes in assessment of threat in the anxiogenic zone and implies the development of a learned form of fear response (Bertoglio and Carobrez, 2000), which has implications for anxiety behavior. Additionally, following this behavioral characterization, we also measured a molecular marker of GABAergic function in the basolateral amygdala (BLA), one of the key hubs of anxiety processing, to gain a window into the molecular underpinnings of changes in anxiety-like behavior following injury.

Our results support the central hypothesis of this study, by demonstrating that early effects on affective behavior following injury can differ from late effects, and that the observed effects can vary depending on the behavior test used. Consequently, they reveal a complex picture regarding the affective consequences of injury, and argue for the need for a standardized and comprehensive approach to study affective outcomes of TBI in animal models.

## Materials and Methods

### Subjects

A total of 42 adult male C57BL6J mice (Jackson Labs, Bar Harbor, ME, USA) were used in the experiments. Mice were split in two groups, CCI and sham. They were housed in colonies of four, with CCI and sham animals mixed in the same cage, in a 12 h light cycle (lights on from 7 am to 7 pm), with constant temperature and humidity (22°C and 40%). Food and water were available *ad libitum*. Animals’ weight was monitored weekly, averaging 32 g. Mice were 6–8 weeks-old at the beginning of experiments and allowed 2 weeks of acclimation before experiments began. Behavioral testing was conducted between 10 am and 5 pm. This study was carried out in accordance with the recommendations of IACUC guidelines. All experimental procedures were approved by Johns Hopkins Animal Care and Use Committee.

### Injury Procedures

Mice were anesthetized with Avertin (2,2,2 tribromoethanol-Sigma, St. Louis, MO, USA) diluted in isotonic saline (500 mg/kg, i.p.). After a midline skin incision, a circular craniotomy was made midway between Bregma and lambda with the medial edge of the craniotomy 0.5 mm lateral to the midline. The mice were then subjected to a moderate to severe (Wakade et al., 2010; Tucker et al., 2017; Wang et al., 2018) CCI injury using a convex impactor tip of 3 mm in diameter. The injury was generated using the following parameters: 4.5 m/s velocity, 1.50 mm depth of penetration and a sustained depression for 150 ms. Mice were given 1 ml saline and 100 μl of 10% Meloxicam subcutaneously. Body temperature was maintained at 37°C with a warming blanket until full recovery from anesthesia (recovery of righting reflex). Sham-operated controls underwent the same surgical procedures with the exception of the traumatic injury. After surgery, mice were kept in individual cages for 72 h and monitored daily, then returned to their home-cages. Survival rate for the surgeries were about 90%. Righting times for both groups for recovery from anesthesia and surgery were similar (CCI: 101 ± 7.09 min; Sham: 103 ± 1 9.17 min). All surviving animals (25 CCI group, 17 sham control group) underwent further behavioral testing following recovery.

### Behavioral Tests and Apparatus

Baseline and post-injury behavioral testing were performed with all mice—TBI and sham—together, in the same order each week. Testing proceeded as follows: animals were brought into the experimental room at least 30 min before the experiments began. They were first tested in the OF, and given at least 2 h to recover before testing on the EZM. Twenty-four hours later, they were brought back to the experimental room and tested in the EPM. Mazes were cleaned with 70% ethanol between each animal. Each mouse was tested twice in each maze before injury. Baseline testing was performed 5–7 days apart, and injury was induced 1 week after the last baseline test. Baseline values were averaged for each animal to calculate their individual baseline level of anxiety.

#### Open-Field Test (OF—Accuscan, Columbus, OH)

The apparatus consisted of a 40.6 cm × 40.6 cm sound-attenuating box, with sensors on the bottom that monitored the animals’ movement. A computer connected to the device recorded time spent in the center and distance traveled. Mice were placed in the center of the field and allowed to freely explore for 20 min. There were two trials before injury and four biweekly trials after injury.

#### Elevated Plus Maze (EPM) Test

Apparatus consisted of two intersecting runways (50 cm × 50 cm × 5 cm), placed at 1 m from the ground. One runway had no walls (two open arms), while the other had 20 cm dark, high walls (two closed arms). Mice were placed in the center of the maze, facing one closed arm, and allowed to explore for 10 min. There were two trials before surgery and four biweekly trials after. A camera placed above the maze recorded the animal’s movement, and trials were analyzed using Ethovision^©^ software. Behavioral metrics monitored were total distance traveled, distance traveled, number of entrances and time spent in each arm.

#### Elevated Zero-Maze (EZM) Test

Apparatus consisted of a circular platform (width: 5 cm, inner diameter: 40 cm), placed at 1 m from the ground and divided in four quadrants. Two opposite quadrants had a 20 cm dark, high walls (closed arm), while the other two had no walls (open arms). Mice were placed facing the entrance of a closed arm, and allowed to explore for 10 min. There were two trials before and four biweekly trials after surgery. A camera placed above the maze recorded the animal’s movement, and trials were analyzed using Ethovision^©^ software. Behavioral metrics monitored were total distance traveled, distance traveled, number of entrances and time spent in each arm.

### Anatomical Metrics and Immunohistochemistry

#### Fixation and Sectioning

Two weeks after behavioral testing was finished, mice were deeply anesthetized (2.5% Avertin, 250 mg/kg body weight, i.p., Sigma, St. Louis, MO, USA) and transcardially perfused with 1 M phosphate buffer saline (PBS, 50 ml) followed by 4% paraformaldehyde (PFA, 100 ml, Sigma, St. Louis, MO, USA). Brains were removed and post-fixed in 4% PFA (50 ml) for 72 h, then transferred to a solution of 30% sucrose in 4% PFA, where they were kept refrigerated until sectioning. Coronal sections (40 μm) of the whole brain were performed using a slide microtome (Leica Microsystems, model CM-1860).

#### Immunohistochemistry

Mounted sections were washed three times for 10 min each wash in 0.1 M PBS and then incubated in blocking solution containing 10% normal goat serum and 0.5% Triton X-100 in PBS for 2 h at room temperature. Slides were then incubated with 1:1,000 of mouse anti-GAD-65/67 (EMD Millipore), 10% normal goat serum, 0.5% Triton X-100, for 72 h at 4°C. The sections were then rinsed three times in PBS, and incubated with the secondary antibody, Alexa Fluor 488 (1:1,000, Abcam), with 5% normal goat serum, 0.5% Triton X-100, for 2 h in room temperature. Sections were washed 3× for 10 min with PBS, and incubated for 30 min with NeuroTrace 640/660 Deep-Red Fluorescent Nissl (Thermo Fisher) and mounted with Vectashield antifade mounting medium (Vector Labs).

#### Imaging for Volumetric Measure

Fluorescent images of the brain in the peri-injury region were taken with an Axiozoom v16 microscope (Carl Zeiss, Germany) using a 10× objective. Volume estimation was performed as described previously (Claus et al., 2010), adopting Cavalieri estimation (Gundersen and Jensen, 1987). Images from four sections were taken for each mouse in each area. We determined the position of each section by using Mouse Brain Atlas coordinates (Paxinos and Franklin, 2004). Sections were located between −0.70 and −2.46 mm Bregma, and they contained the lesioned area in the injured mice or equivalent position in sham controls. BLA images were taken between −1.22 and 2.18 mm Bregma. Images were pre-processed using Fiji software (Schindelin et al., 2012). The hemispheric and BLA areas were outlined using Fiji on the ipsilateral and contralateral sides of the four brain sections, volume was calculated as the sum of the areas multiplied by the distance between sections (300 μm). The ipsilateral volume was normalized to the contralateral volume to estimate the extent of hemispheric volume loss (lesion) and change in BLA volume.

#### Imaging for Immunohistochemistry

Fluorescent images of the BLA were taken with a Zeiss LSM 700 microscope (Carl Zeiss, Germany) using 20× and 40× objectives. Four bilateral immunofluorescent labeled sections were taken in each mouse (eight images per animal), sections were approximately 200 μm apart. Images were pre-processed using Fiji (Schindelin et al., 2012). Number and intensity of GAD puncta were calculated using a custom MATLAB analysis package (IMFLAN3D; Tai et al., 2007). Here, a “punctum” was defined as a cluster of “connected” pixels identified in an unbiased manner using the MATLAB function *bwlabeln* with the “eight-connected neighborhood” criterion.

### Statistical Analysis

**Figure 1:**
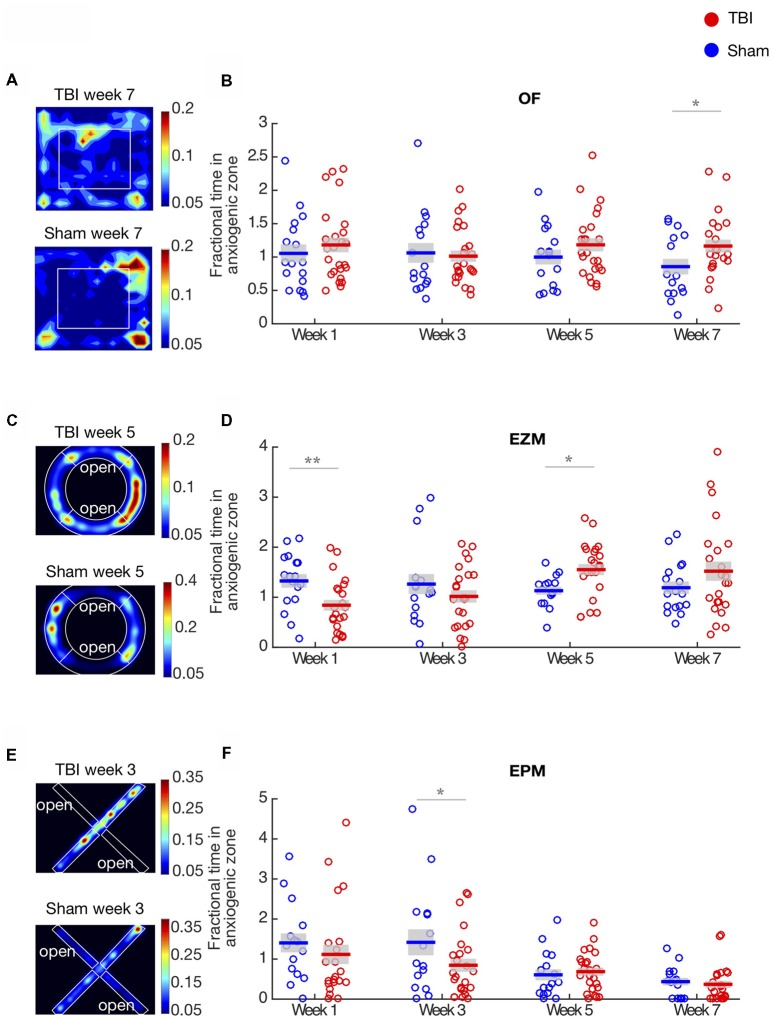
Traumatic brain injury (TBI) causes long-term effects on affective behaviors. **(A,C,E)** Representative heat maps of TBI and Sham animals in the open field (OF), elevated zero maze (EZM) and elevated plus maze (EPM), respectively. Warmer colors represent that the animals spent more time on that zone. **(B)** Proportion of time in the center of the OF arena. Each circle represents one mouse. Horizontal bars denote means; shaded regions denote SEM. There is a main effect of injury (repeated two-way ANOVA, *p* < 0.01) and anxiety significantly decreases on week 7. **(D)** Proportion of time in the open arm of the EZM; conventions as in **(B)**. There is an interaction between time and injury (repeated two-way ANOVA, *p* < 0.01). Anxiety is significantly increased on week 1 and decreased on week 5. **(F)** Proportion of time in the open arm of the EPM; conventions as in **(B)**. There is a main effect of injury and time (repeated two-way ANOVA, *p* < 0.01). Anxiety significantly increases on week 3. Panels **(B,D,E)** show data are from *n* = 25 mice in TBI condition, and *n* = 17 mice in sham condition after removal of outliers at each time point (“Materials and Methods” section); the number of outliers at any time point for any condition did not exceed four mice; *p < 0.05, **p < 0.01 by *post hoc* *t*-test with false discovery rate (FDR) correction.

**Figure 2:**
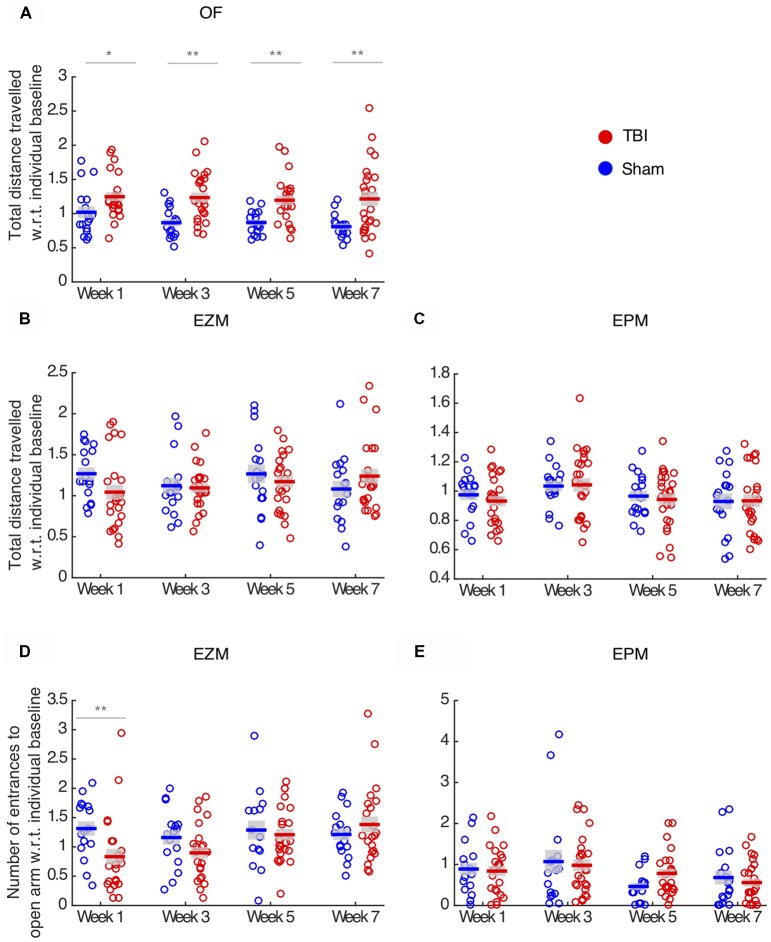
TBI effects vary across behavioral assays and metrics. **(A)** Total distance traveled in the OF. There is a main effect of injury (repeated two-way ANOVA, *p* < 0.01), and TBI animals travel more at all time-points compared to sham controls. **(B,C)** Total distance traveled in the EZM and EPM, respectively. There is no effect of injury in locomotion in these assays. **(D)** Number of entrance to the open arm in the EZM. There is a main effect of injury (repeated two-way ANOVA, *p* < 0.05) and TBI animals present fewer entrance on week 1 than sham controls. **(E)** Number of entrances to the open arm in the EPM. There is no effect of injury. In all panels, each circle represents one mouse. Horizontal bars denote means; shaded regions denote SEM. Data from *n* = 25 mice in TBI condition, and *n* = 17 mice in sham condition are shown after removal of outliers at each time point (“Materials and Methods” section); the number of outliers at any time point for any condition did not exceed three mice; *p < 0.05, **p < 0.01.

**Figure 3:**
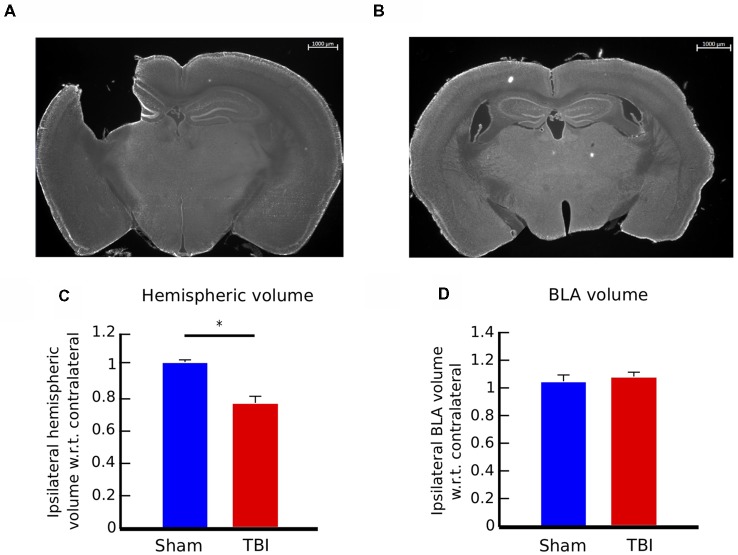
Controlled cortical impact (CCI) causes consistent injury across animals and no volumetric change in the basolateral amygdala (BLA). **(A)** 12× panoramic coronal sections stained with Fluoro Nissl, representing the lesion in the cortex and hippocampus for TBI animals and **(B)** intact areas in sham controls. **(C)** Hemispheric volume in TBI and sham animals. There is a significant reduction in the ipsilateral hemisphere volume of injured animals, compared to sham controls (*t*-test, *p* < 0.05). **(D)** Volumetric measure of the BLA. Injury does not cause volumetric change in the BLA of TBI animals. Bar graphs in **(C,D)** show mean ± SEM of data from *n* = 10 mice in the TBI condition and *n* = 10 mice in the sham condition after removal of outliers (“Materials and Methods” section); the number of outliers did not exceed one mouse for any condition; *p < 0.05.

**Figure 4:**
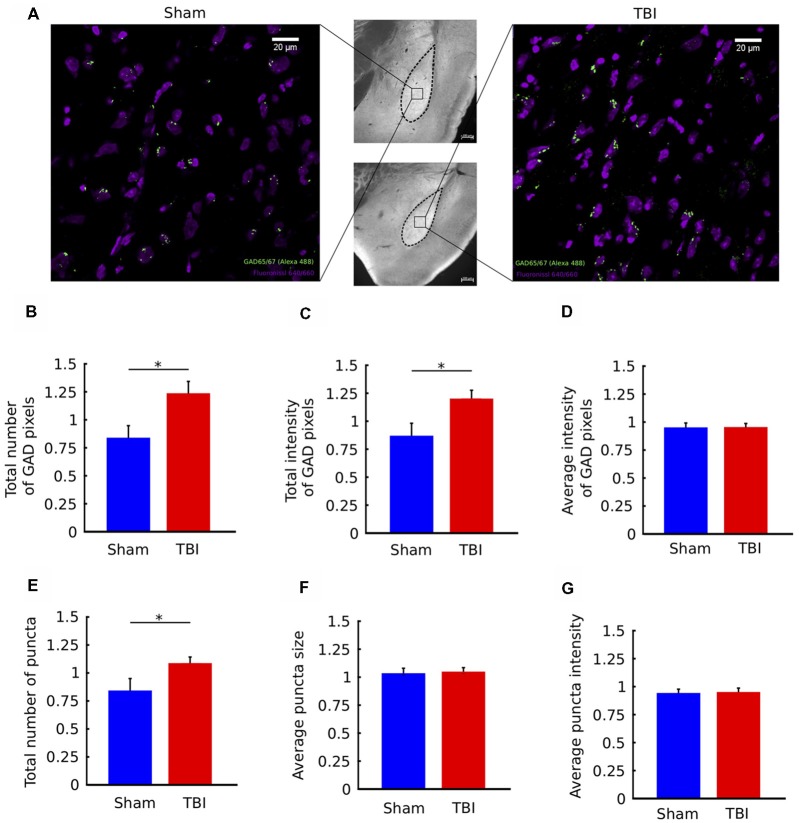
TBI is associated with upregulation of GAD immunostaining in the ipsilateral amygdala. **(A)** Middle column: 10× images of histological sections from a sham animal (top) and TBI animal (bottom). Left/Right columns: 63× views of the indicated portions of the sections. Fluoro Nissl staining in purple and GAD65/67 staining in green. **(B–G)** Quantification of GAD65/67 expression in the ipsilateral and contralateral BLA in four sections per animal. Metrics in **(B–G)** are reported as ratios of ipsilateral to contralateral values for each animal. In injured animals, there was a significant increase in the total number of GAD pixels **(B)** and the total intensity of GAD pixels **(C)**, but not the average intensity of GAD pixels **(D)**. In addition, there was an increase in the total number of GAD “puncta” (clusters of contiguous GAD pixels **(E)**, but not in the average size of puncta **(F)** or average intensity of puncta **(G)**. All bar graphs show mean ± SEM of data from *n* = 10 mice in the TBI condition and *n* = 10 mice in the sham condition after removal of outliers (“Materials and Methods” section); the number of outliers did not exceed two mice for any; *p < 0.05 (two-tailed *t*-test).

For the behavioral tasks, a two-way unbalanced repeated measure ANOVA was performed after standard methods of outlier exclusion in MATLAB were applied. Data shown (at each time point in Figures 1, 2, and in Figures 3, 4) represent distributions obtained after removal of outliers following a standard procedure in MATLAB: samples that deviated from the median by more than fac*interquartile range were deemed outliers (average value of fac used = 1.5). *Post hoc* *t*-tests were performed whenever ANOVA indicated a significant effect, and *p*-values were corrected by a false discovery rate (FDR) test. For hemispheric volume, the normalized ipsilateral volumes (ipsilateral/contralateral) were compared among groups using *t*-tests and FDR correction. For GAD65/67 puncta analysis, the mean of each group was compared using *t*-test with FDR correction. Individual baseline data was averaged across the two pre-injury sessions to determine the mean value for each animal and assay. Post-injury behavioral data was plotted normalized to each animal’s individual baseline: behavioral metrics at each time-point was divided by the animal’s baseline value. Values greater than one indicate the mouse’s anxiety level decreased in comparison to its baseline, whereas values smaller than one indicate increase in anxiety. This approach allows us to measure how anxiety changed for each individual animal because of the injury. Normalized values are presented as mean ± standard error (SEM). All statistical tests were performed using MATLAB (Mathworks Inc., Natick, MA, USA). Statistical analysis was considered significant for *p* < 0.05.

## Results

### Effects of TBI on Anxiety-Like Behaviors Exhibit a Complex Trajectory

We determined the long-term effects of TBI on affective outcomes in a battery of innate anxiety behavioral tests following exposure to a CCI injury. Mice were tested in the OF, EZM and EPM tests. These tasks exploit the animals’ natural conflict between seeking protection and exploring a novel environment. All mice were tested twice in the behavior tests before surgery, and these were averaged to determine each animal’s baseline level of anxiety. Post-injury or sham surgery, they were then tested on weeks 1, 3, 5 and 7. Anxiety was measured by the change in proportion of time they spent in the anxiogenic zone in each assay, as well as number of entrances to the open arm (EZM and EPM). Values at each time point are normalized to each animal’s baseline; this approach allowed us to determine how the anxiety level of each animal changed due to the injury. We controlled for possible locomotion deficits, by measuring total distance traveled in all three mazes.

Examples of heat maps representing the proportion of time TBI and sham mice spent in each zone in the OF arena on week 7 are illustrated in Figure 1A. In this test, we found an overall effect of injury across the population, tested by an unbalanced repeated two-way ANOVA (*F*_(1,3)_ = 4.04, *p* < 0.01, Figure 1B). Injured mice spent significantly more time in the center of the arena on week 7 (*post hoc* *t*-test with FDR correction, *p* = 0.02), but we did not observe difference between groups in the other time-points. These results indicate that the main effect of injury was driven by the strong decrease in anxiety TBI mice presented on week 7.

In order to compare the effect of injury across different behavioral assays, we measured anxiety-like behaviors in the EZM and EPM tests. Heat maps illustrating the proportion of time mice spent in each type of arm are shown in Figures 1C,E. In the EZM, there was no main effect of injury in the proportion of time spent in the open arm (unbalanced repeated two-way ANOVA, *F* = 0.002, *p* = 0.96, Figure 1D). However, we found a strong interaction effect between time and injury (unbalanced repeated two-way ANOVA, *F*_(1,3)_ = 4.24, *p* < 0.01). TBI mice display increased anxiety on week 1 (*post hoc* *t*-test with FDR, *p* < 0.01) and decreased anxiety on week 5 (*post hoc* *t*-test with FDR correction, *p* = 0.01), as measured by time spent in the open arms. Interestingly, in both the OF and EZM, we observed a decrease in anxiety-like behaviors as compared to sham controls, on week 7 and 5, respectively, which suggests a delayed effect of the injury. There were no differences between groups in baseline, prior to TBI (Supplementary Figure 1).

In addition to the OF and EZM tests, we measured anxiety in the EPM. Figure 1F shows the proportion of time mice spent in the open arm in this assay. We found a main effect of injury, tested by an unbalanced repeated two-way ANOVA (*F*_(1,3)_ = 4.44, *p* = 0.03), and a strong effect of time (unbalanced repeated two-way ANOVA, *F*_(1,3)_ = 9.18, *p* < 0.01). *Post hoc* *t*-test with FDR correction showed that TBI mice spent significantly less time in the open arm on week 3, indicating increased anxiety (*p* < 0.05). We observed that both in the EZM and EPM tests, there was an increase of anxiety-like behaviors on earlier time-points: on week 1 for the EZM and week 3 in the EPM. Across the 3 behavioral assays, our results indicate that the effects of TBI on anxiety-like behaviors are time- and task-dependent: generally, TBI led to an early increase and late decrease in anxiety-like behaviors.

As an additional metric of anxiety, we measured number of entrances to the open arm in the EZM and EPM tests. Injured mice did not differ from sham on number of entrances in the EPM (unbalanced repeated two-way ANOVA, *F*_(1,3)_ = 0.25, *p* = 0.61, Figure 2D). In the EZM, we observed an effect of injury in the number of entrances to the open arm (unbalanced repeated two-way ANOVA, *F*_(1,3)_ = 5.10, *p* = 0.02, Figure 2E), and TBI mice presented significantly fewer entrances on week 1 (*post hoc* *t*-test with FDR correction, *p* = 0.01), which is consistent with the decreased anxiety observed in the proportion of time in the open arm on week 1 in this maze.

To ensure that anxiety metrics were not affected by locomotion deficits, we measured total distance traveled in each maze. In the OF, TBI led to hyperactivity throughout all time points (unbalanced repeated two-way ANOVA, *F*_(1,3)_ = 36.69, *p* < 0.01, *post hoc* *t*-test with FDR correction, *p* > 0.05, Figure 2A). Injury had no locomotion effect on the EZM (unbalanced repeated two-way ANOVA, *F*_(1,3)_ = 0.56, *p* = 0.45, Figure 2B) and EPM tests (unbalanced repeated two-way ANOVA, *F*_(1,3)_ = 1.23, *p* = 0.26, Figure 2C). Since TBI and sham did not differ in terms of total distance traveled in the EZM, we concluded that the reduced number of entrances to the open arm in this maze reflects the increase in anxiety those mice presented on week 1. Finally, we concluded that the effects in anxiety-like behaviors were not confounded by locomotion deficits.

Our behavioral results indicated that the effects of TBI in anxiety-like behaviors are complex. Although injury did not affect locomotion in the EPM and EZM tests, the effect on anxiety metrics varied across time-points and assays. By comparing different assays, we identified that in the first few weeks after injury, mice had a significant increase in anxiety-like behaviors. This effect, however, inverted after about a month post-injury. After this point, TBI mice display decreased anxiety-like behaviors as compared to sham controls.

### Injury Was Consistent Across Mice

Considering the complex effects of injury, we asked if differences in the extent of injury across mice could be a confounding factor. Because we were interested in the long-term effects of injury, we sacrificed the mice on week 9 post-injury or sham surgery, obtained brain sections and quantified hemispherical volumes as an anatomical metric of the extent of injury.

Coronal brain sections of an injured mouse illustrating the extent of the injury to the cortex and hippocampus, as well as a brain section in the same region of control mouse are presented in Figures 3A,B. We determined the extent of the injury by measuring the hemispheric area of the peri-injury site, on the ipsilateral and contralateral sides of four brain sections per mouse, located between −0.70 and −2.46 mm Bregma, and multiplied the sum of the areas for each side by the distance between sections (300 μm; *n* = 10 for each group). The ipsilateral hemispheric volume of each brain was then normalized to its contralateral side. There is no effect of injury on the contralateral side (data not shown). The injury caused a reduction of the ipsilateral hemispheric volume of approximately 20%, compared to control mice (Figure 3C, two-tailed *t*-test, *p* < 0.01). The extent of the injury was consistent among TBI mice, as shown by the standard error of the mean (SEM_TBI_ = ±0.04, SEM_sham_ = ±0.01).

### Injury Did Not Cause a Volumetric Change in the BLA

We proceeded to identify potential neural correlates of the long-term behavioral outcomes. Past work has demonstrated that injury can cause volumetric changes in brain areas distal from the site of injury. Motivated by this, we hypothesized that the affective behavioral changes could correlate with anatomical changes in the BLA, a key brain area involved in the control of emotional responses. To test this hypothesis, we measured volumetric changes in the BLA on week 9.

Volume of the BLA was estimated by measuring area of four sections on each side per mouse, and multiplying the area of the BLA by the distance between sections (300 μm). BLA sections were located between −1.22 and −2.18 mm Bregma. The ipsilateral volume was then normalized to its contralateral side (*n* = 10 for each group). There is no effect of injury on the contralateral side (data not shown). There was no significant difference in BLA volume between injured and sham mice, as shown in Figure 3D (two-tailed *t*-test, *p* = 0.7). These results indicate that the changes in anxiety-like behaviors were not correlated with changes in volume in the BLA.

### Neural Marker: Immunostaining Indicates Upregulation of GAD Ipsilaterally

Next, we assessed functional neural correlates of the long-term affective outcomes of injury. To investigate whether the observed late decrease in anxiety had a molecular correlate, we examined the strength of inhibitory signaling in the BLA. To this end, we performed GAD immunostaining by targeting GAD65/67, which are two enzymes expressed in the brain and involved in the synthesis of GABA. GAD67 is expressed equally through the cell body, while GAD65 is mainly found in nerve terminals (Asada et al., 1996). We chose to target GAD because it has been demonstrated that GABA plays a crucial role in anxiety disorders (Kalueff and Nutt, 2007). We quantified several metrics of GAD expression, such as number and intensity of GAD puncta. Ipsilateral values were normalized to contralateral for each mouse.

Figure 4A shows coronal sections zoomed-in on the BLA, with labeling of cell bodies and GAD puncta for a sham and a TBI mouse. There was an overall increase in the total number of GAD-stained pixels in the ipsilateral BLA of injured mice, measured by a two-tailed *t*-test (*p* = 0.03, Figure 4B). There was also an overall increase in total GAD signal intensity (*p* = 0.01, Figure 4C). Notably, the average intensity of GAD-stained pixels was not different between groups (*p* > 0.05, Figure 4D). Together, these results indicate that injury causes an increase in the number of GAD-stained pixels, but not in the intensity (brightness) of individual pixels.

To understand if there were effects on spatial clustering of GAD-stained pixels, we next analyzed the properties of groups of contiguous (or connected) GAD-pixels, called “puncta” (see “Materials and Methods” section). Compared to individual pixels, which can be contaminated by noise, puncta are more likely to represent functional signal. We found that there was an increase in the number of GAD puncta following injury (Figure 4E, *p* = 0.04), but no change in the average size of puncta or the average intensity of puncta (*p* > 0.05, Figures 4F,G). In other words, consistent with the results from individual pixels, GAD puncta are not larger or brighter, they are greater in number following injury. Together, the immunostaining results show an upregulation of GAD immunostaining in the ipsilateral BLA of TBI mice.

## Discussion

Human TBI has a complex pathology, and studies show that, among the many outcomes of injury, patients are at a higher risk of suffering from anxiety disorders (Rao and Lyketsos, 2002; Moore et al., 2006; Scholten et al., 2016). Reports on the prevalence of anxiety following injury are variable: pooled long-term prevalence is reported at 36%, according to a recent review (Scholten et al., 2016), but some studies suggest prevalence between 11% and 70% (Rao and Lyketsos, 2002). One unsolved issue is our lack of understanding about the neural mechanisms of injury that may increase a patient’s chance of developing an anxiety disorder. Animal models of TBI are valuable to address this problem, for their ability to control for injury parameters and to allow us to measure behavioral changes and neural markers in well-controlled experiments. However, a complex, and at times contradictory, picture has emerged from various animal studies. It is important to comprehensively study these models over long time frames, to develop an understanding of TBI pathophysiology and its impact on affective behavior.

In this study, we adopt a well-established and highly controlled mouse injury model (CCI), and test mice in three assays of innate anxiety over a 7-week time-course, to evaluate how the evolving consequences of TBI impacts affective behavioral function. We demonstrate that the effects of moderate to severe TBI on anxiety-like behaviors are complex and long lasting. Additionally, with this behavioral characterization as a basis, we also measured a molecular marker of GABAergic function in the BLA, one of the key hubs of anxiety processing, and we demonstrated that there is an upregulation of GAD staining at 9 weeks post-injury.

Early after TBI, injury caused a significant increase in anxiety-like behavior measured in the EPM and EZM tests, consistent with several studies that show increased anxiety acutely after injury to the murine brain (Meyer et al., 2012; Almeida-Suhett et al., 2014; Tucker et al., 2017). However, no such effect was observed in the OF test. This is consistent with Sierra-Mercado et al. (2015), who tested the effect of CCI in mice 1 week after injury, and found no change in anxiety as measured by the OF test. Interestingly, a few other studies are able to measure this early increase in anxiety in the OF test as well (Wagner et al., 2007; Almeida-Suhett et al., 2014; Tucker et al., 2017). This inconsistency could be explained by differences in severity of injury (Tucker et al., 2017) or rodent model (Wagner et al., 2007; Almeida-Suhett et al., 2014). However, the difference between effects observed among behavioral tests in our study—within the same group of mice at the same time points—underlines the complexity of measuring anxiety-like behavior in animal models. An important potential implication of this finding is that different assays of anxiety do not always measure the same aspect of anxiety-like behaviors. A comprehensive approach to behavioral testing following TBI is therefore imperative to draw useful conclusions. In order to study the progression of the anxiety response following injury, it is necessary to employ a repeated testing model. One concern with repeated measurement for behavioral tests of anxiety is that of potential habituation and learning. While repeated testing has been shown to have effects on anxiety tests in many studies (Griebel et al., 1993; Adamec et al., 2005; Walf and Frye, 2007), Bertoglio and Carobrez (2000) suggest that the decreased exploration of open arm in the EPM could be related to a qualitative shift from unconditioned to a learned form of fear response. Thus, what is generally considered habituation or learning in the testing arena, is likely a change in the acquired fear response underlying the expression of anxiety-like behavior. Changes in the sham group over time suggest that some of these factors do play a role in this study. Sham mice presented habituation to the OF and EPM, observed in the decrease in proportion of time spent in the anxiogenic zone over time post-surgery on the OF and EPM, as well as in decrease over time in the total distance traveled in the OF. These results are consistent with previous literature, which has demonstrated that repeated exposure to the OF and EZM lead to adaptation to the apparatus measured by decreased exploration of the anxiogenic zone and decreased overall locomotion (Rodgers and Shepherd, 1993; Treit et al., 1993; Logue et al., 1997; Bolivar et al., 2000).

While this study does not rule out these factors in the testing arena, that effect is consistent between both sham and injured groups, and differences observed between groups can be attributed to the TBI. Changes in the expression of anxiety behavior can therefore be interpreted as a deficit in the learned fear response due to re-exposure, a deficit in learning about the context of the anxiogenic zone, or as an increase in risk taking behavior. Direct assays measuring changes in fear learning, such as acoustic startle, might help to parse out these changes over time.

The affective response to TBI is not limited to the early time points, but evolves over time, reflecting the fact that neural mechanisms of injury evolve over time (Werner and Engelhard, 2007). Five weeks after TBI, injured mice show significantly less anxiety-like behaviors. At this stage, the mice showed an increase in exploration of the open arm in the EZM test, as compared to sham controls. The mice also display an increased tendency to explore the center anxiogenic zone in the OF test. Curiously, a similar increase in exploration of the open arm is not observed in the EPM test. Our findings are consistent with findings of decreased anxiety-like behavior in the EZM test at later time points after injury (Jones et al., 2008; Thau-Zuchman et al., 2019), as well as decreased anxiety in the OF test in rats exposed to lateral fluid percussion at 1 and 3 months post-injury (Jones et al., 2008). Interestingly, several studies have found decreased anxiety in the EPM as well, tested in mice following CCI at 20 days (Washington et al., 2012) and rats tested in a closed-head model at between 12 and 30 days (Pandey et al., 2009; Schwarzbold et al., 2010). This difference could also be explained by the fact that we use repeated measurements of behavior in the EPM. Similarly, Ajao et al. (2012) measured an initial increase in anxiety like behavior after juvenile rats were exposed to CCI, which appears to reverse at later time points.

Injury also caused increased locomotion in the OF throughout all time-points, consistent with other studies (Jones et al., 2008; Pandey et al., 2009; Tucker et al., 2017). This suggests that the observed hyperactivity is independent of the early increase and later decrease in anxiety-like behavior. These findings are consistent with human studies where hyperactivity has been reported following pediatric TBI (Max et al., 1998) and impulsivity has been reported following TBI in adults (James et al., 2014; Kocka and Gagnon, 2014).

Several neural mechanisms underlying anxiety changes following injury have been investigated (Schwarzbold et al., 2010; Ajao et al., 2012; Elder et al., 2012; Meyer et al., 2012; Reger et al., 2012; Almeida-Suhett et al., 2014; Sönmez et al., 2015; Tucker et al., 2017). Of particular interest is the BLA, which has been linked to changes in anxiety-like behaviors. Causal manipulations of projections from and to the BLA directly alter anxiety-like behaviors (Scott et al., 1997; Amano et al., 2011; Tye et al., 2011; Felix-Ortiz et al., 2013; Hong et al., 2014), indicating that this area is an important hub for emotional responses in the brain. In addition, signaling by GABA, the major inhibitory neurotransmitter in the nervous system, is greatly implicated in psychiatric disorders such as anxiety and depression (Lydiard, 2003; Nemeroff, 2003; Möehler, 2006; Kalueff and Nutt, 2007). Infusion of GABA_A_ receptor antagonist into the amygdala increases anxiety (Sanders and Shekhar, 1995), whereas GABA agonist infusion into the BLA reduces anxiety (Bueno et al., 2005), indicating that the inhibitory balance within the amygdala drives changes in anxiety. In previous TBI studies, it has been shown that reduced GABAergic inhibition and increase in number of neurons in the BLA correlate with enhanced anxiety in rodents early after injury (Meyer et al., 2012; Almeida-Suhett et al., 2014).

In this study, while the volumetric measures of the peri-injury area indicate a reduction in ipsilateral hemispheric volume as expected (Bueno et al., 2005; Washington et al., 2012; Almeida-Suhett et al., 2014), there is no significant difference in volumetric measures of the BLA between injured mice and sham controls. However, GAD immunostaining is upregulated in the ipsilateral BLA at 9 weeks following injury, indicating increased GABAergic inhibition within this area. This increase is correlated with late decreases in anxiety-like behavior of the injured animals at later time points. Our results are consistent with causal, GABA_A_ agonist infusion experiments in the BLA (Chen et al., 2003). Almeida-Suhett et al. (2014) observed TBI-induced decrease in GABAergic inhibition and an increase in anxiety-like behavior at early time points after injury, whereas this study shows the opposite effects at later time points. Thus, our results regarding GABA signaling are in line with previous work and extend our understanding about the longer-term effects of injury.

In summary, we demonstrate a time-dependent reversal in the course of affective behavioral response following traumatic injury to the mouse brain: an early increase followed by a late decrease in anxiety, with the latter being correlated with an increase in GABAergic inhibition in the BLA. In addition to revealing a complex affective trajectory, results support the hypothesis that the lack of consensus across past studies (Jones et al., 2008; Pandey et al., 2009; Washington et al., 2012) on the effects on anxiety outcomes following injury may be the result of the variability in injury models used, behavioral assays of anxiety chosen and time-points at which assessments were made. Consequently, they highlight the need for the use of a reproducible model of injury as well as the use of multiple assays and time-points within future studies. Such an approach can provide a consistent foundation for investigating the neural mechanisms underlying affective outcomes of TBI and the development of therapeutic strategies.

## Data Availability

The datasets generated for this study are available on request to the corresponding author.

## Author Contributions

HA and SM designed the study. JP carried out experiments and analyzed the data. All authors contributed to the interpretation of the results and writing of the manuscript.

## Conflict of Interest Statement

The authors declare that the research was conducted in the absence of any commercial or financial relationships that could be construed as a potential conflict of interest.
