# Supplementary material for: Long-Term Effects of Traumatic Brain Injury on Anxiety-Like Behaviors in Mice: Behavioral and Neural Correlates
Source: Front Behav Neurosci. 2019 Jan 23;13:6. doi: 10.3389/fnbeh.2019.00006 (PMC6351473; doi:10.3389/fnbeh.2019.00006)
Supplement: Supplementary file 1 [file Data_Sheet_1.PDF]

Supplementary Figure 1: baseline behavior metrics

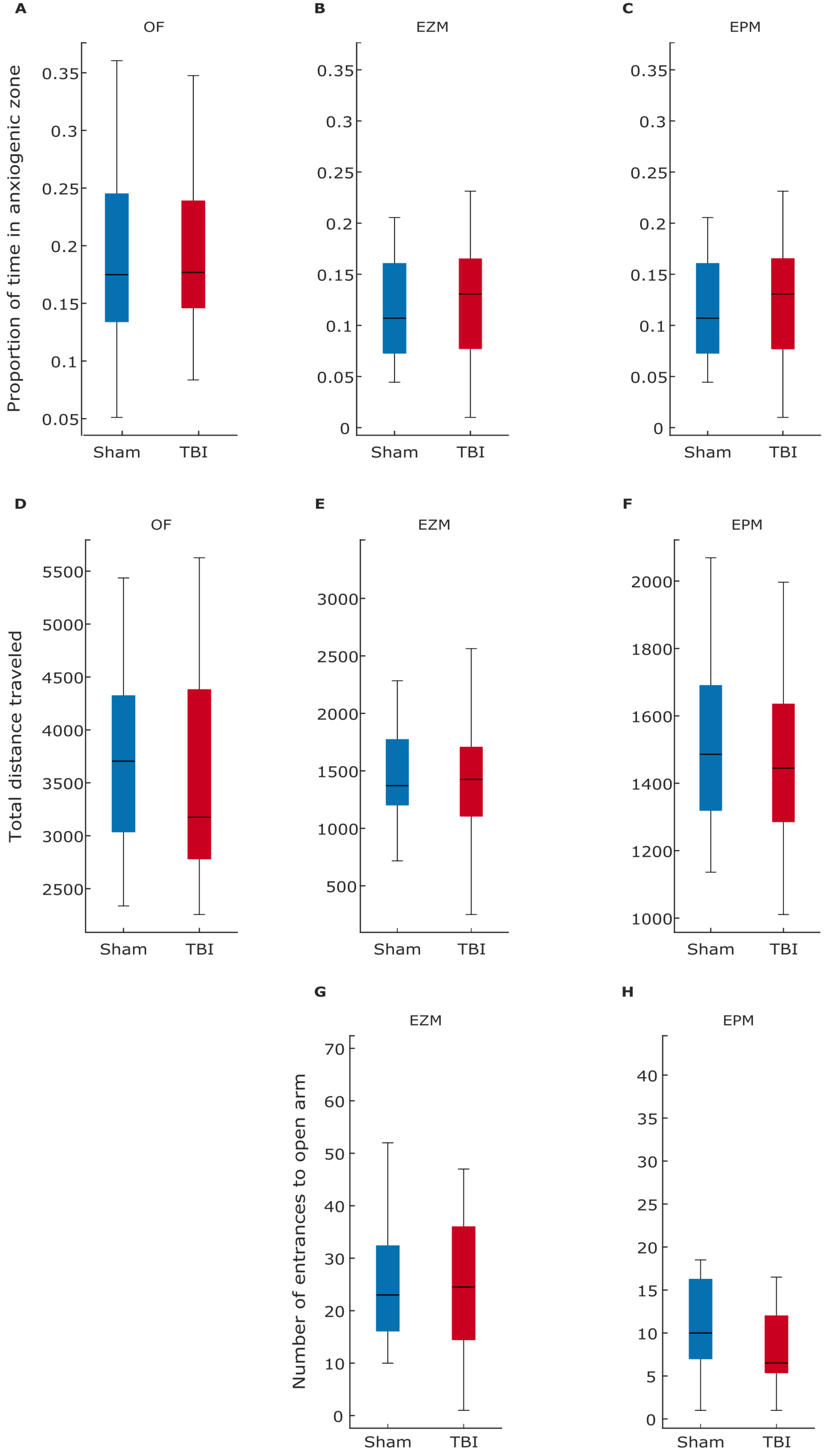

Supplementary Figure 1: Baseline behavioral metrics for sham and TBI animals. A) Proportion of time in the center of the OF arena; B) Proportion of time in the open arm in the EZM; C) Proportion of time in the open arm in the EPM. D) Total distance traveled in the OF arena. E) Total distance traveled in the EZM; F) Total distance traveled in the EPM; G) Number of entrances to the open arm in the EZM; H) Number of entrances to the open arm in the EPM. There was no difference between groups during the baseline in any behavioral metric, tested by t-tests with FDR correction. TBI: n=25, sham: n=17.
